# Recent hospitalization and risk of antidepressant initiation in people with Parkinson’s disease

**DOI:** 10.1186/s12877-022-03698-w

**Published:** 2022-12-17

**Authors:** Iida Hämäläinen, Miia Tiihonen, Sirpa Hartikainen, Anna-Maija Tolppanen

**Affiliations:** 1 Kuopio Research Centre of Geriatric Care, University of Eastern Finland, Kuopio, Finland; 2 School of Pharmacy, University of Eastern Finland, P.O. Box 1627, 70211 Kuopio, Finland

**Keywords:** Parkinson’s disease, Antidepressant, Hospitalization, Register-based study

## Abstract

**Background:**

People with Parkinson’s disease (PD) are more likely to be hospitalized and initiate antidepressant use compared to people without PD. It is not known if hospitalization increases the risk of antidepressant initiation. We studied whether a recent hospitalization associates with antidepressant initiation in people with PD.

**Methods:**

A nested case-control study within the nationwide register-based FINPARK cohort which includes community-dwelling Finnish residents diagnosed with PD between years 1996 and 2015 (*N* = 22,189) was conducted. Initiation of antidepressant use after PD diagnosis was identified from Prescription Register with 1-year washout period (cases). One matched non-initiator control for each case was identified (*N* = 5492 age, sex, and time since PD diagnosis-matched case-control pairs). Hospitalizations within the 14 day-period preceding the antidepressant initiation were identified from the Care Register for Health Care.

**Results:**

The mean age at antidepressant initiation was 73.5 years with median time since PD diagnosis 2.9 years. Selective serotonin reuptake inhibitors (48.1%) and mirtazapine (35.7%) were the most commonly initiated antidepressants. Recent hospitalization was more common among antidepressant initiators than non-initiators (48.3 and 14.3%, respectively) and was associated with antidepressant initiation also after adjusting for comorbidities and use of medications during the washout (adjusted OR, 95% CI 5.85, 5.20–6.59). The initiators also had longer hospitalizations than non-initiators. PD was the most common main discharge diagnosis among both initiators (54.6%) and non-initiators (28.8%). Discharge diagnoses of mental and behavioral disorders and dementia were more common among initiators.

**Conclusions:**

Hospitalisation is an opportunity to identify and assess depressive symptoms, sleep disorders and pain, which may partially explain the association. Alternatively, the indication for antidepressant initiation may have led to hospitalisation, or hospitalisation to aggravation of, e.g., neuropsychiatric symptoms leading to antidepressant initiation.

**Supplementary Information:**

The online version contains supplementary material available at 10.1186/s12877-022-03698-w.

## Background

Parkinson’s disease (PD) is the second most common neurodegenerative disorder whose prevalence increases with age [1]. Diagnosis of PD is based on presence of motor symptoms but it is estimated that at the time of motor symptoms appear, more than half of the dopaminergic neurons are lost [2]. The symptoms of PD are heterogeneous involving also clinically important non-motor symptoms, and the pathology of PD involves several regions of the nervous system and various neurotransmitters [2, 3]. The non-motor neuropsychiatric symptoms include, for example, depression, anxiety, sleep disorders, psychosis and in later stage cognitive deficits.

The prevalence of neuropsychiatric symptoms in persons with PD is higher than in the general older population. According to a meta-analysis, prevalence of clinically significant depressive symptoms in people with PD was 35% [4]. However, that meta-analysis included studies conducted in different study populations including patients from early to late stage of PD. The study settings were also varied, and higher prevalence of depressive symptoms was reported in studies from hospital inpatient settings than in population-based, general practice, outpatient, or nursing home settings [4]. Nevertheless, nearly all people with PD suffer from neuropsychiatric symptoms during the course of the disease [5].

Neuropsychiatric symptoms occur often concurrently and have a significant negative impact on the quality of life and daily functioning in people with PD. [6–9] Due to these symptoms, the use of psychotropic drugs is common in people with PD. [10–12] People with PD are more likely to initiate antidepressant use than those without PD, [12, 13] despite limited evidence on efficacy and safety of antidepressants in people with PD. [14] However, the indications for initiation of antidepressants are unclear but likely to be multifaceted including mood and sleep disorders as well as pain.

People with PD are more likely to be hospitalized, and have longer hospital stays than people without PD. [15–17] According to a recent population-based cohort study which investigated rate and reasons for hospitalization among people with PD compared to the general population, psychotic symptoms or hallucinations and dementia were significantly more common reasons for hospital admission among people with PD. [17] Hospitalizations, particularly prolonged hospitalizations, increase the risk of neuropsychiatric symptoms in older patients [18]. In addition, incorrect dopaminergic medication and infections during hospitalization may decline motor functioning in PD. [19, 20]

We investigated whether a recent hospitalization was associated with antidepressant initiation among people with PD. To examine the possible reasons for antidepressant initiation, we also investigated the discharge diagnoses.

## Methods

### Study setting

The study is based on the nationwide register-based Finnish study on Parkinson’s disease (FINPARK) which includes 22,189 community-dwelling Finnish residents who received a clinically confirmed diagnosis of PD between years 1996 and 2015.

Study participants were identified from the Special Reimbursement Register maintained by the Social Insurance Institution of Finland (SII). The register provides the data of special reimbursements for medicines due to chronic diseases, such as PD. The diagnosis of PD entitles to a special reimbursement of antiparkinson drugs when the medical statement is evaluated by the SII. The medical statement for special reimbursement includes anamnesis of the patient and description of the characteristic clinical features of PD including, for example, bradykinesia, rigidity, and tremor. The reimbursement of antiparkinson drugs is granted to the patient if predefined criteria are fulfilled and diagnosis is confirmed by a neurologist. According to the Finnish Current Care Guideline the diagnosis of PD is based on the United Kingdom Parkinson’s Disease Society Brain Bank criteria which have obtained good accuracy of diagnoses [21, 22]. PD is, however, challenging to diagnose in the early stage [23]. Antiparkinson drugs are also used for other conditions than PD which entitle for the same special reimbursement if the efficiency of drugs is demonstrated. Thus, those who did not have ICD-10 code for PD (G20), were less than 35 years old on the PD diagnosis date, or had diagnoses whose symptoms may be confused with PD, or which may indicate false diagnosis of PD (−/+ 2 years of the PD diagnosis date) were excluded. The exclusion diagnoses included e.g. secondary parkinsonism, dystonia, multi-system degeneration, Huntington disease, Alzheimer’s disease, and multiple sclerosis. The exclusion and inclusion criteria have been described in detail earlier [12].

### Study design

A case-control study with antidepressant initiators as cases was conducted. Persons with history of schizophrenia, or bipolar disorder at least 5 years before PD diagnosis were excluded from the study from both initiators and non-initiators as the study focuses on the use of antidepressants for neuropsychiatric symptoms of PD. In addition, persons with a newly diagnosed cancer within 2 years before the index date were excluded. Definitions for the exclusion criteria are listed in Supplementary Table 1.

**Fig. 1:**
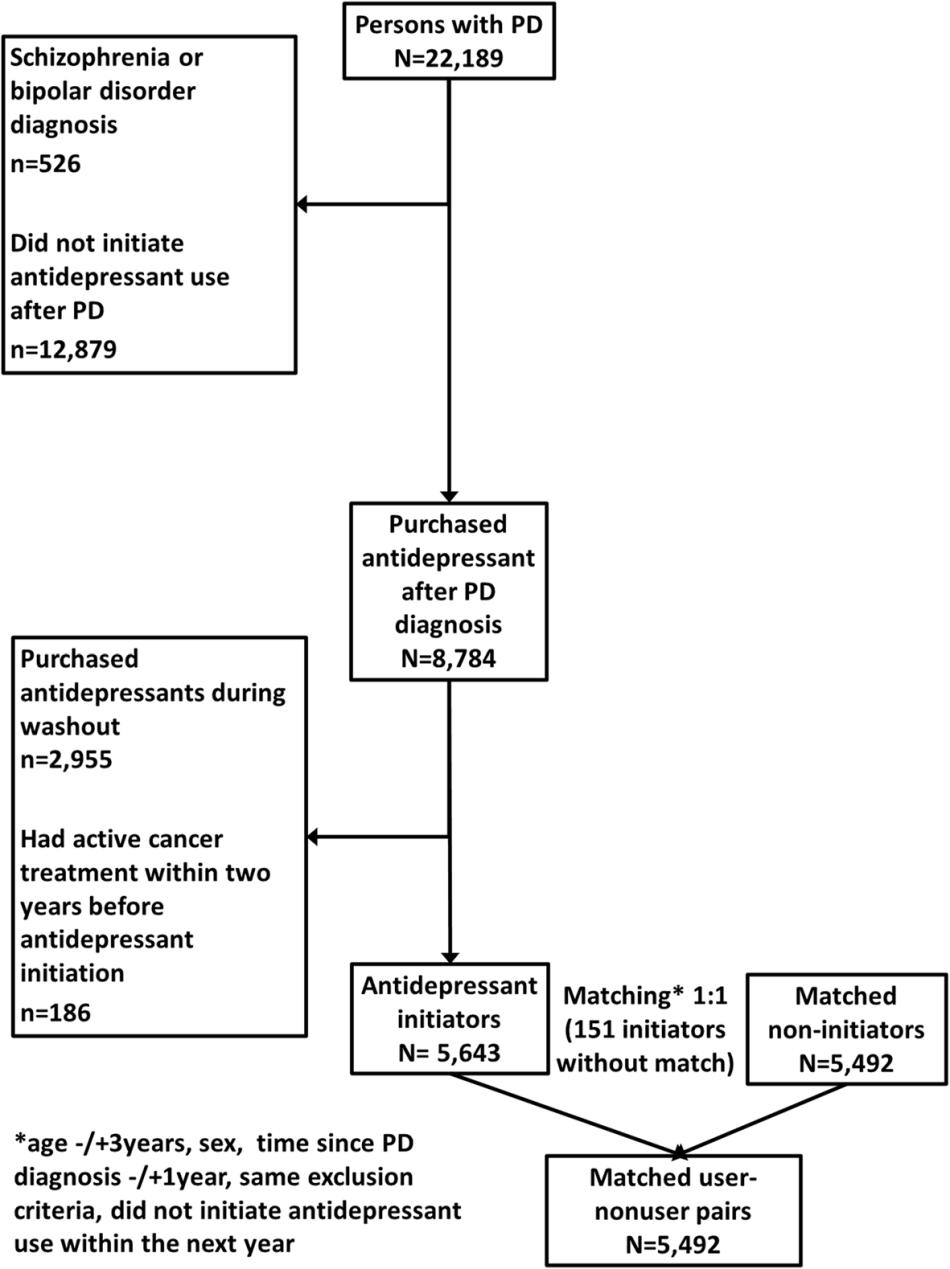
Formation of the study sample

Antidepressant drug initiation after PD diagnosis was identified with a 1-year washout period (*n* = 5643, Fig. 1) using Prescription Register which includes data of all reimbursed drug purchases of community-dwelling people outside a hospital or other institutional care facilities since 1995. We used the washout period to exclude those persons who purchased antidepressants shortly (less than 1 year) after PD diagnosis but had in fact already, relatively recently purchased antidepressants before PD diagnosis. Antidepressants were defined in accordance with the Anatomical Therapeutic Chemical (ATC) classification system code N06A [24].

A non-initiator control, matched by age (+/− 3 years), sex, and time since PD diagnosis (+/− 365 days), was identified for each initiator with antidepressant initiation date as the index date. The number of matched case-control pairs was 5492. Non-matched initiators were excluded from the analyses (*n* = 151). The same exclusion criteria were used for cases and controls.

Information on comorbidities and use of drugs during the washout period were collected from national registers (Supplementary Table 1). Use of the antiparkinson drugs (N04), antipsychotics (N05A, excluding lithium N05AN01 and prochlorperazine N05AB04), benzodiazepines and related drugs (N05BA, N05CD, N05CF), opioids (N02A), acetylcholinesterase inhibitors (N06DA) and memantine (N06DX01) were obtained from the Prescription Register. Comorbidities, i.e. asthma, chronic obstructive pulmonary disease (COPD), diabetes, and cardiovascular diseases until the matching date were recognized from the Special Reimbursement Register.

Hospitalization data were collected from the Care Register for Health Care, including the main discharge diagnosis as ICD-10 codes, the length of hospital stay and the admission place which was categorized as central/university or regional/municipal hospital. Recent hospitalization was determined as a discharge from hospital a maximum of 14 days before the index date.

Permission for the data use was obtained from the register maintainers. Data were collected from the registers by the register maintainers utilizing personal identification numbers. According to Finnish legislation, no Ethics Committee approval was required for the study since study participants were not contacted and data were de-identified.

### Statistical analyses

Statistical analyses were performed using Stata (MP14.0). The Chi-squared test was used to compare categorical variables between the groups. Normally distributed continuous variables were tested with two sample t-test and asymmetrically distributed variables with nonparametric median test (Mann-Whitney U-test). The associations of recent hospitalization and duration of hospitalization with antidepressant initiation were assessed with conditional logistic regression. This method controls for matching and therefore controls for age, sex and time since PD diagnosis. In addition, the results were adjusted for comorbidities and medication use during the washout period.

## Results

The mean age of the study population at the time of antidepressant initiation was 73.5 years and the median time since PD diagnosis was 2.9 years (Table 1). Approximately half (52.3%) of the initiators were women while more than 60% of the study population were men. Use of benzodiazepines and related drugs, opioids, antipsychotics, acetylcholinesterase inhibitors, and antiparkinson drugs during washout was more common among antidepressant initiators than non-initiators. Initiators also had more cardiovascular diseases, asthma/COPD, and diabetes and were more likely to have been hospitalized within 14 days before the index date (48.3% of the initiators compared to 14.3% of non-initiators).

**Table 1:** Characteristics of antidepressant initiators and matched non-initiators with PD

Characteristic	Initiators (*n* = 5492)	Non-initiators (*n* = 5492)	*P* Value
Age at index date^a^, mean (95% CI)	73.5 (73.3–73.8)	73.6 (73.4–73.9)	Matched
Time since PD diagnosis^a^, median (IQR)	2.9 (1.1–5.5)	2.8 (1.1–5.5)	Matched
Sex, n (%)			Matched
Women	2873 (52.3)	2873 (52.3)	
Men	2619 (47.7)	2619 (47.7)	
Hospitalization within 14 days before the index date^a^, n (%)	2653 (48.3)	788 (14.3)	< 0.001
Comorbidities, n (%)			
Cardiovascular diseases	2356 (42.9)	1285 (23.4)	< 0.001
Asthma/COPD	442 (8.1)	243 (4.4)	< 0.001
Diabetes	610 (11.1)	309 (5.6)	< 0.001
PD medication use, n (%)			
Any medication for PD	5202 (94.7)	4815 (87.7)	< 0.001
Dopaminergic medication for PD	5178 (94.3)	4793 (87.3)	< 0.001
Dopa and dopa derivatives	4536 (82.6)	3882 (70.7)	< 0.001
Dopamine agonists	2304 (42.0)	2246 (40.9)	0.26
Monoamine oxidase B inhibitors	1961 (35.7)	2003 (36.5)	0.41
Other dopaminergic agents	290 (5.3)	222 (4.0)	0.002
Other medication use, n (%)			
Opioids	860 (15.7)	504 (9.2)	< 0.001
Antipsychotics	571 (10.4)	355 (6.5)	< 0.001
Benzodiazepines and related drugs	1831 (33.3)	835 (15.2)	< 0.001
Acetylcholinesterase inhibitors (AChEI)	389 (7.1)	289 (5.3)	< 0.001
Memantine	62 (1.1)	52 (1.0)	0.35
AChEI/memantine	406 (7.4)	307 (5.6)	< 0.001

^a^the date of initiator starting antidepressant use

Recently hospitalized persons were six times more likely to initiate antidepressants (unadjusted OR 5.93, 95% CI 5.32–6.62) and the association remained after adjusting for comorbidities and medication use during washout (Table 2). Longer hospital stays were associated with increased risk.

**Table 2:** Odds ratios and 95%CI for the association between recent hospitalization, duration of hospitalization and antidepressant initiation

Recent hospitalisation	Unadjusted OR, 95%CI^a^	Comorbidity-adjusted OR%, 95CI^b^	Comorbidity- and medication-adjusted OR%, 95CI^c^
No (reference	1.00 (reference)	1.00 (reference)	1.00 (reference)
Yes	5.93 (5.32–6.62)	5.85 (5.23–6.55)	5.85 (5.20–6.59)
The length of hospital stay			
1–7 days	1.00 (reference)	1.00 (reference)	1.00 (reference)
8–14 days	1.76 (1.14–2.74)	1.71 (1.08–2.69)	1.82 (1.12–2.98)
15–60 days	2.82 (1.81–4.38)	2.95 (1.87–4.66)	3.74 (2.28–6.15)
61 days or longer	6.40 (2.55–16.07)	5.34 (2.07–13.79)	7.41 (2.64–20.83)

^a^conditional logistic regression, accounts for age, sex, time since PD

^b^conditional logistic regression, adjusted for cardiovascular disease, diabetes, stroke, asthma or chronic obstructive pulmonary disease

^c^conditional logistic regression, adjusted for comorbidities and use of antiparkinson medication, antipsychotics, benzodiazepines and related drugs, opioids and antidementia medications

Selective serotonin reuptake inhibitors (SSRIs) (48.1%) and mirtazapine (35.7%) covered over 80% of the initiations (Supplementary Table 2). The initiation of serotonin and norepinephrine reuptake inhibitors (SNRIs), tricyclic antidepressants (TCAs) and other antidepressants was less common. Nearly all initiated with monotherapy as only 28 people (0.5%) initiated two antidepressants. No clinically meaningful differences in initiated antidepressants between recently hospitalized and non-hospitalized initiators were observed (Table 3).

**Table 3:** Characteristics of hospitalized and non-hospitalized antidepressant initiators with PD

Characteristic	Hospitalized (*n* = 2653)	Non-hospitalized (*n* = 2839)	*P* value
Age at index date ^a^, mean (95% CI)	73.2 (72.8–73.5)	73.9 (73.5–74.2)	0.007
Time since PD diagnosis, median (IQR)	3.0 (1.2–5.6)	2.7 (1.1–5.4)	0.006
Sex, n (%)			0.18
Women	1363 (51.4)	1510 (53.2)	
Men	1290 (48.6)	1329 (46.8)	
Comorbidities, n (%)			
Cardiovascular diseases	1118 (42.1)	1238 (43.6)	0.27
Asthma/COPD	234 (8.8)	208 (7.3)	0.04
Diabetes	309 (11.7)	301 (10.6)	0.22
PD medication use, n (%)			
Any medication for PD	2511 (94.7)	2691 (94.8)	0.82
Dopaminergic medication for PD	2502 (94.3)	2676 (94.3)	0.94
Dopa and dopa derivatives	2191 (82.6)	2345 (82.6)	0.99
Dopamine agonists	1205 (45.4)	1,09 (38.7)	< 0.001
Monoamine oxidase B inhibitors	930 (35.1)	1031 (36.3)	0.33
Other dopaminergic agents	142 (5.4)	148 (5.2)	0.82
Other medication use, n (%)			
Opioids	486 (18.3)	374 (13.2)	< 0.001
Antipsychotics	286 (10.8)	285 (10.0)	0.37
Benzodiazepines and related drugs	839 (31.6)	992 (34.9)	0.009
Acetylcholinesterase inhibitors (AChEI)	151 (5.7)	238 (8.4)	< 0.001
Memantine	23 (0.9)	39 (1.4)	0.08
AChEI/memantine	158 (6.0)	248 (8.7)	< 0.001
Antidepressant initiation, n (%)			
Selective serotonin reuptake inhibitor	1266 (47.7)	1374 (48.4)	0.62
Mirtazapine	996 (37.5)	964 (34.0)	0.006
Serotonin and norepinephrine reuptake inhibitor (SNRI)	157 (5.9)	130 (4.6)	0.026
Tricyclic antidepressant (TCA)	155 (5.8)	233 (8.2)	0.001
Other ^b^	98 (3.7)	147 (5.2)	0.008
Initiated 2 antidepressants	19 (0.7)	9 (0.3)	

^a^the date of initiator starting antidepressant use

^b^Mianserin, moclobemide, bupropion, trazodone, reboxetine, vortioxetine, agomelatine

SSRIs were the most frequently initiated antidepressant class in both groups (47.7% of recently hospitalized and 48.4% of non-hospitalized initiators), followed by mirtazapine (37.5% of hospitalized and 34.0% of non-hospitalized initiators). Initiated SSRIs were mainly citalopram and escitalopram which equally covered 44% of initiations among recently hospitalized and 41.9% of initiations among non-hospitalized initiators. Use of dopamine agonists and opioids were more common in hospitalized initiators, whereas use of benzodiazepines and related drugs, and acetylcholinesterase inhibitors was more common among other initiators.

Compared to non-initiators with recent hospitalization, the initiators with recent hospitalization had longer length of hospital stays, and were more often discharged from municipal or regional hospital (Table 4). The most common discharge diagnosis of the recent hospitalization was PD (ICD-10 code G20) among both initiators (54.6%) and non-initiators (28.8%). Among initiators, PD diagnosis was followed by the diseases of circulatory (6.3%), and musculoskeletal system and connective tissues (4.4%). Mental or behavioral disorders (F04-F99) were recorded as a main discharge diagnosis for 4.0% of hospitalized antidepressant initiators and 0.5% of hospitalized non-initiators. Dementia (F00-F03, G30) was also slightly more common discharge diagnosis among initiators (3.6%) than non-initiators (1.3%). In addition, hospitalized initiators used more likely antiparkinson drugs, opioids, and benzodiazepines and related drugs during washout period (Table 5). Cardiovascular diseases and diabetes were more common in hospitalized initiators (42.1 and 11.7%, respectively) in comparison to hospitalized non-initiators (23.5 and 4.8%, respectively).

**Table 4:** Hospitalization-related characteristic of recently hospitalized initiators and non-initiators with PD

Characteristic	Hospitalized Initiators (*n* = 2653)	Hospitalized non-initiators (*n* = 788)	*P* value
The length of hospital stay, n (%)			< 0.001
1–7 days	1637 (61.7)	626 (79.4)	
8–14 days	371 (14.0)	89 (11.3)	
15–60 days	460 (17.3)	61 (7.7)	
61 or more	185 (7.0)	12 (1.5)	
Admission place, n (%)			< 0.001
Central/university hospital	1529 (57.6)	589 (74.8)	
Regional/municipal hospital	1124 (42.4)	199 (25.3)	
Main discharge diagnosis group by ICD-10 codes, n (%)			< 0.001
Parkinson’s disease (G20)	1449 (54.6)	227 (28.8)	
Diseases of the circulatory system (I)	166 (6.3)	70 (8.9)	
Diseases of the musculoskeletal system and connective tissue (M)	116 (4.4)	40 (5.1)	
Mental and behavioral disorders (F04-F99)	107 (4.0)	4 (0.5)	
Symptoms, signs and abnormal findings, not elsewhere classified (R)	102 (3.8)	36 (4.6)	
Dementia (F00-F03, G30)	95 (3.6)	10 (1.3)	
Injury, poisoning and certain other consequences of external causes (S + T)	83 (3.1)	24 (3.0)	
Diseases of the nervous system, excluding G20, G30 (G)	62 (2.3)	15 (1.9)	
Congenital malformations/ factors influencing health status and contacts with health services (Q + Z)	60 (2.3)	35 (4.4)	
Diseases of the genitourinary system (N)	57 (2.1)	39 (4.9)	
Diseases of the respiratory system (J)	43 (1.6)	24 (3.0)	
Diseases of the eye and adnexa, ear and mastoid process (H)	42 (1.6)	60 (7.6)	
Diseases of the digestive system (K)	35 (1.3)	25 (3.2)	
Malignant neoplasms (C)	32 (1.2)	26 (3.3)	
Certain infectious and parasitic diseases (AB)	24 (0.9)	13 (1.6)	
Endocrine, nutritional and metabolic diseases (E)	22 (0.8)	12 (1.5)	
Other neoplasms/ diseases of blood and blood-forming organs and certain disorders involving the immune mechanism (D)	16 (0.6)	21 (2.7)	
Diseases of the skin and subcutaneous tissue (L)	14 (0.5)	7 (0.9)

**Table 5:** Characteristic of recently hospitalized initiators and non-initiators with PD

Characteristic	Hospitalized Initiators (*n* = 2653)	Hospitalized non-initiators (*n* = 788)	*P* value
Age at index date^a^, mean (95% CI)	73.2 (72.8–73.5)	73.5 (72.8–74.1)	0.48
Time since PD diagnosis, median (IQR)	3.0 (1.2–5.6)	2.8 (1.2–5.6)	0.11
Sex, n (%)			0.04
Women	1363 (51.4)	438 (55.6)	
Men	1290 (48.6)	350 (44.4)	
Comorbidities, n (%)			
Cardiovascular diseases	1118 (42.1)	185 (23.5)	< 0.001
Asthma/COPD	234 (8.8)	50 (6.4)	0.03
Diabetes	309 (11.7)	38 (4.8)	< 0.001
PD medication use, n (%)			
Any medication for PD	2511 (94.7)	718 (91.1)	< 0.001
Dopaminergic medication for PD	2502 (94.3)	717 (91.0)	0.001
Dopa and dopa derivatives	2191 (82.6)	595 (75.5)	< 0.001
Dopamine agonists	1205 (45.4)	353 (44.8)	0.76
Monoamine oxidase B inhibitors	930 (35.1)	301 (38.2)	0.11
Other dopaminergic agents	142 (5.4)	45 (5.7)	0.70
Other medication use, n (%)			
Opioids	486 (18.3)	116 (14.7)	0.020
Antipsychotics	286 (10.8)	77 (9.8)	0.42
Benzodiazepines and related drugs	839 (31.6)	132 (16.8)	< 0.001
Acetylcholinesterase inhibitors (AChEI)	151 (5.7)	45 (5.7)	0.98
Memantine	23 (0.9)	11 (1.4)	0.19
AChEI/memantine	158 (6.0)	48 (6.1)	0.89

^a^the date of initiator starting antidepressant use

## Discussion

Our findings demonstrate that a recent hospitalization was associated with an increased risk of antidepressant initiation in community-dwelling people with PD since nearly half of the initiators were hospitalized within 14 days before the index date compared to 14% of the non-initiators. Overall, comorbid diseases, use of antiparkinson and other observed medications, longer hospital stay and regional/municipal hospital as admission place were associated with increased risk of antidepressant initiation. SSRIs were the most frequently initiated antidepressant class.

PD was the most frequently recorded main discharge diagnosis in both groups which could imply aggravation of PD symptoms. Our finding is in accordance to a previous study that found exacerbation of motor and non-motor symptoms of PD are the main underlying reason for hospital admissions in people with PD. [17] Although a considerable proportion of discharge diagnoses in our study were other than PD, these admissions may still be related to PD. For example, mental and behavioral disorder and dementia diagnoses that were more common among initiators than non-initiators may also reflect symptoms of PD.

According to previous studies, other common reasons for hospital admissions in people with PD are cardiovascular events, infections, such as urinary and respiratory tract infections, falls and fractures which are fairly consistent with our results considering that our study population consisted of antidepressant initiators and matched non-initiators [17, 25]. These diagnosis categories were among the most common ones in both initiators and non-initiators, with being slightly more common in non-initiators.

The median time since PD diagnosis between initiators and non-initiators was similar (2.9 years) as this was a matching criterion. However, the use of dopa and dopa derivatives during washout was more common among antidepressant initiators which could indicate more advanced disease among them compared to non-initiators. On the other hand, the prevalence of dopaminergic PD medication was quite high in both groups (94.3% in initiators and 87.3%) in noninitiators, and the difference was relatively small (7%).

Depression has been associated with severity of PD and dementia, but not with age at PD onset or duration of PD. [26] Another study investigated neuropsychiatric symptoms and use of psychotropic medication during the first 5 years since PD diagnosis [27]. In that study, neuropsychiatric symptoms, and use of antiparkinson medication, but also the use of antidepressants and benzodiazepines and related drugs became more common during the study period. Use of antidepressants and benzodiazepines and related drugs was also common at baseline, while use of antipsychotics and acetylcholine inhibitors and memantine (0.0–5.1%), was rare at baseline and remained low throughout the 5-year period [27].

In our study about 25% (5643/22,189) initiated antidepressant use after PD diagnosis and nearly half of them were recently hospitalized. It could be that depression or depressive symptoms were identified during the hospitalization, leading to initiation of pharmacotherapy. This is supported by a previous study where clinically significant depressive symptoms were present in 35% of people with PD. [4] According to the same meta-analysis, prevalence of major depressive disorder was 17%. Minor depression was present in 22% and dysthymia in 13%. Prevalence varied in included studies from 2.7 to 90% likely due to differences between study population, study methods, diagnosis and definition of depression, and statistical methods. Moreover, onset or aggravation of depressive and other neuropsychiatric symptoms could lead to hospitalization, or hospitalization could trigger those symptoms.

Antidepressant treatment is considered in moderate to severe depression or in milder cases if non-pharmacological methods have not been successful [28]. Non-motor fluctuations related to wearing off of dopaminergic medication could manifest as low mood, apathy, or fatigue which could be confused to symptoms of depression. In these cases, optimization of dopaminergic medication can be beneficial. While management of depressive and other neuropsychiatric symptoms is important, evidence on efficacy of antidepressants is limited in people with PD due to lack of controlled trials. In the context of neurodegeneration, depression may not respond similarly to medication developed for endogenous depression since underlying mechanism for PD depression is complex [13, 14, 29]. However, multiple antidepressant classes, such as SSRIs, SNRIs and TCAs are potentially efficacious in management of depression in PD, but trial results are inconsistent, and superiority of antidepressants over placebo is not always demonstrated [13, 14, 30].

SSRIs were the most frequently initiated antidepressants in our study and they are commonly the first line treatment due to their safety profile [28]. TCAs are considered as potentially inappropriate medications in older adults due to anticholinergic (including e.g. cognitive decline, constipation, dizziness) and sedative properties as well as orthostatic hypotension [31]. Out of the different antidepressants, SSRIs are most strongly associated with hyponatremia and gastrointestinal bleedings [32–37]. Additionally, SSRIs have been related with extrapyramidal symptoms and worsening of tremor in people with PD. [30] Antidepressants are also associated with cardiac effects; SSRIs and TCAs can cause QT prolongation, with greater effect on TCAs [38]. Antidepressants increase the risk of falls which is already increased in people with PD due to antiparkinson medication and primary symptoms of PD. [39, 40]

In addition to depression, antidepressants are prescribed for other indications, such as anxiety, sleep disorders and pain which all are common symptoms among people with PD. [7, 41–43] Pain in PD is commonly nociceptive but could be also attributed to neuropathic or diverse sources [43]. In our study population, opioids were used by every sixth initiator during washout which might indicate moderate or severe pain. TCAs and SNRIs are considered a first line pharmacotherapy for neuropathic pain, [44] which could partly explain antidepressant initiation. However, initiations of TCAs and SNRIs were not common in our study.

Benzodiazepines and related drugs were used by every third antidepressant initiator, which may indicate sleep and anxiety disorders. Insomnia is one of the most common non-motor symptoms together with depression, and longer disease duration as well as dopaminergic medication increase the risk [7, 45]. Thus, the high prevalence of mirtazapine in our study could be partly explained by the use of low-dose mirtazapine for the treatment of insomnia [41, 42]. Mirtazapine has sedative properties mainly through histamine receptor blocking [46]. Furthermore, previous study showed that about one-third of people with PD is suffering from anxiety, most commonly generalized anxiety disorder, and phobias [47]. In addition, depressive symptoms and anxiety disorders often occur concomitantly and anxiety disorders are an important indication of SSRIs.

Depression and other neuropsychiatric symptoms seem to be poorly recognized in persons with PD since depressive symptoms could overlap with primary symptoms of PD. [48] Better awareness and assessment of neuropsychiatric symptoms would promote more effective care [48, 49]. Management of these symptoms is important since they decrease quality of life and functioning in people with PD. [6–9] As depression seems to be underrecognized, hospitalization might be opportunity to recognize depressive symptoms, and thus contribute to increased likelihood of antidepressant initiations after admission. Diagnoses of depressive symptoms might not occur as discharge diagnosis in this study since only main discharge diagnoses were observed. Neuropsychiatric symptoms can lead to hospitalization, thus would be important to prevent hospital admissions by managing those symptoms [50]. However, initiation of antidepressant should be considered carefully, and hospitalization should not increase inappropriate drug use. Cognitive-behavioral therapy is effective management of depression and anxiety in PD and recommended to consider as a treatment option [51].

The main strength of this study was the use of the large nationwide registers with systemically collected data, enabling the identification of community-dwellers with clinically confirmed diagnosis of PD in Finland, and assessment of antidepressants dispensed in community-dwelling settings purchases. We had also comprehensive information on main discharge diagnoses of hospitalized patients. On the other hand, we lacked information whether purchased antidepressants were used, although purchased drugs represent drug exposure better than prescribed drugs [52]. Since drug use during hospital care was not recorded in the Prescription Register, we lacked information whether antidepressants were initiated during hospitalization. Consequently, we did not know if antidepressant was prescribed during hospital stay or after hospitalization. Indications for the antidepressant drug prescriptions, information whether non-pharmacological treatment was attempted, and initiation of antidepressant was carefully assessed were lacking, thus we could not evaluate the appropriateness of treatment. Other limitation of this study was lack of information on the severity of PD and presence or severity of neuropsychiatric symptoms.

In conclusion, a recent hospitalization and longer hospital stay were associated with antidepressant initiation in community-dwelling people with PD. Further investigation is needed to clarify accurate indications for initiations of antidepressants. Our findings suggest that motor and non-motor symptoms of PD are the leading cause for hospitalization in this population. Hospitalization could be also an opportunity to identify and assess depressive symptoms, sleep disorders and pain.

## Supplementary Information

**Additional file 1: Supplementary Table 1. **Description of antidepressant use, exclusion criteria and covariates. **Supplementary Table 2.** Initiated antidepressants on drug and group level.

## Data Availability

The data that support the findings of this study are available from the corresponding author but restrictions apply to the availability of these data, and so they are not publicly available. Data are however available from the authors upon reasonable request and with permission of the register maintainers.

## Abbreviations

ATC: Anatomical therapeutic chemical classification
COPD: Chronic obstructive pulmonary disease
FINPARK: Finnish Study on Parkinson’s disease
PD: Parkinson’s disease
SII: Social Insurance Institution of Finland
SNRI: Serotonin–norepinephrine reuptake inhibitor
SSRI: Serotonin reuptake inhibitor
TCA: Tricyclic antidepressant
