# Supplementary material for: Recent hospitalization and risk of antidepressant initiation in people with Parkinson’s disease
Source: BMC Geriatr. 2022 Dec 17;22:974. doi: 10.1186/s12877-022-03698-w (PMC9758789; doi:10.1186/s12877-022-03698-w)
Supplement: Supplementary file 1 — Additional file 1: Supplementary Table 1. Description of antidepressant use, exclusion criteria and covariates. Supplementary Table 2. Initiated antidepressants on drug and group level. [file 12877_2022_3698_MOESM1_ESM.docx]

# Supplementary material for

# Recent hospitalization and risk of antidepressant initiation in people with Parkinson’s disease

Authors:Iida Hämäläinen, Miia Tiihonen, Sirpa Hartikainen, Anna-Maija Tolppanen

Corresponding author: Anna-Maija Tolppanen, School of Pharmacy, University of Eastern Finland, P.O. Box 1627, 70211 Kuopio, Finland, [anna-maija.tolppanen@uef.fi](mailto:anna-maija.tolppanen@uef.fi)

Supplementary Table 1. Description of antidepressant use, exclusion criteria and covariates.

| Variable | Data sources and coding | Years |
| --- | --- | --- |
| Antidepressant use (outcome) | Prescription register: ATC code N06A | 1995-2015 (use after PD diagnosis with one-year washout) |
| Newly diagnosed cancer  (exclusion) | Cancer register | Within two years before the index date |
| Schizophrenia, other chronic psychoses or bipolar disorder  (exclusion) | Care register for health care ICD-10 F20-29;  [ICD-9](https://www.sciencedirect.com/topics/medicine-and-dentistry/icd-9) 295, 297, 298, 3010 and 3012;  [ICD-8](https://www.sciencedirect.com/topics/medicine-and-dentistry/icd-8) 295, 297, 298, 29999, 30100 and 30120)  ICD-10 F30-31; ICD-9 2962, 2963, 2964 and 2967; ICD-8 29610, 29620, 29630, 29688 and 29699 | 1972-five years before PD diagnosis |
| Asthma/chronic obstructive pulmonary disease (COPD) | Special reimbursement register code 203 | From 1995 to the beginning of the follow-up |
| Cardiovascular disease | Special reimbursement register code 201, 205, 206, 207, 213, 280 | From 1995 to the beginning of the follow-up |
| Diabetes | Special reimbursement register code 103 | From 1995 to the beginning of the follow-up |
| Any medication for Parkinson’s disease | Prescription register: ATC code N04  (includes anticholinergic, dopaminergic and other antiparkinson drugs) | During washout period (within one year before the index date) |
| Dopaminergic medication for Parkinson’s disease | Prescription register: ATC code N04B excluding amantadine N04BB01 | During washout period (within one year before the index date) |
| Dopa and dopa derivatives | Prescription register: ATC code N04BA | During washout period (within one year before the index date) |
| Dopamine agonists | Prescription register: ATC code N04BC | During washout period (within one year before the index date) |
| Monoamine oxidase B inhibitors | Prescription register: ATC code N04BD | During washout period (within one year before the index date) |

| Other dopaminergic agents | Prescription register: ATC code N04BX | During washout period (within one year before the index date) |
| --- | --- | --- |
| Opioids | Prescription register: ATC code N02A | During washout period (within one year before the index date) |
| Antipsychotics | Prescription register: ATC code N05A excluding lithium N05AN01 and prochlorperazine N05AB04 | During washout period (within one year before the index date) |
| Benzodiazepines and related drugs | Prescription register: ATC code N05BA, N05CD, N05CF | During washout period (within one year before the index date) |
| Acetylcholinesterase inhibitor | Prescription register: ATC code NO5BA, N05CD, N05CF | During washout period (within one year before the index date) |
| Memantine | Prescription register: ATC code NO5BA, N05CD, N05CF | During washout period (within one year before the index date) |

Supplementary Table 2. Initiated antidepressants on drug and group level.

| Antidepressant (ATC code) | All initiators  n (%), N=5,492 | Hospitalized initiators  n (%), N=2,653 | Non-hospitalized initiators, n (%), N=2,839 |
| --- | --- | --- | --- |
| **Selective serotonin reuptake inhibitor** | 2,640 (48.1) | 1,266 (47.7) | 1,374 (48.4) |
| Citalopram (N06AB04) | 1,472 (26.8) | 709 (26.7) | 763 (26.9) |
| Escitalopram (N06AB10) | 887 (16.2) | 460 (17.3) | 427 (15.0) |
| Sertraline (N06AB06) | 119 (2.2) | 37 (1.4) | 82 (2.9) |
| Fluoxetine (N06AB03) | 103 (1.9) | 43 (1.6) | 60 (2.1) |
| Paroxetine (N06AB05) | 45 (0.8) | 15 (0.6) | 30 (1.1) |
| Fluvoxamine (N06AB08) | 14 (0.3) | 2 (0.1) | 12 (0.4) |
| **Mirtazapine (N06AX11)** | 1,960 (35.7) | 996 (37.5) | 964 (34.0) |
| **Serotonin and norepinephrine reuptake inhibitor** | 287 (5.2) | 157 (5.9) | 130 (4.6) |
| Duloxetine (N06AX21) | 170 (3.1) | 95 (3.6) | 75 (2.6) |
| Venlafaxine (N06AX16) | 115 (2.1) | 62 (2.3) | 53 (1.9) |
| Milnacipran (N06AX17) | 2 (0.04) | 0 (0.0) | 2 (0.1) |
| **Tricyclic antidepressant (TCA)** | 388 (7.1) | 155 (5.8) | 233 (8.2) |
| Amitriptyline (N06AA09) | 218 (4.0) | 95 (3.5) | 123 (4.3) |
| Doxepin (N06AA12) | 120 (2.2) | 43 (1.6) | 77 (2.7) |
| Nortriptyline (N06AA10) | 29 (0.5) | 12 (0.5) | 17 (0.6) |
| Trimipramine (N06AA06) | 17 (0.3) | 4 (0.2) | 13 (0.5) |
| Clomipramine (N06AA04) | 4 (0.1) | 1 (0.04) | 3 (0.1) |
| **Other** | 245 (4.5) | 98 (3.7) | 147 (5.2) |
| Mianserin (N06AX03) | 189 (3.4) | 67 (2.5) | 122 (4.3) |
| Moclobemide (N06AG02) | 15 (0.3) | 6 (0.2) | 9 (0.3) |
| Bupropion (N06AX12) | 12 (0.2) | 10 (0.4) | 2 (0.1) |
| Trazodone (N06AX05) | 11 (0.2) | 6 (0.2) | 5 (0.2) |
| Reboxetine (N06AX18) | 8 (0.2) | 5 (0.2) | 3 (0.1) |
| Vortioxetine (N06AX26) | 7 (0.1) | 3 (0.1) | 4 (0.1) |
| Agomelatine (N06AX22) | 3 (0.05) | 1 (0.04) | 2 (0.1) |
| **Initiated 2 antidepressants** | 28 (0.5) | 19 (0.7) | 9 (0.3) |
